# Help Received for Perceived Needs Related to Mental Health in a Montreal (Canada) Epidemiological Catchment Area

**DOI:** 10.3390/ijerph121013016

**Published:** 2015-10-16

**Authors:** Marie-Josée Fleury, Jean-Marie Bamvita, Guy Grenier, Jean Caron

**Affiliations:** 1 Department of Psychiatry, McGill University; Douglas Mental Health University Institute; Montreal, PQ H4R 1R3, Canada; E-Mail: jean.caron@douglas.mcgill.ca; 2 Montreal Addiction Rehabilitation Centre—University Institute (CRDM-IU), Montreal, PQ H2M 2E8, Canada; 3 Douglas Hospital Research Centre, Montreal, PQ H4H 2R3, Canada; E-Mails: jean-marie.bamvita@douglas.mcgill.ca (J.-M.B.); guy.grenier@douglas.mcgill.ca (G.G.)

**Keywords:** epidemiological study, help received, PNCQ, counselling, medication, information

## Abstract

This study sought to identify variables associated with help received in terms of information, medication, counselling and total help received (including other needs) among 571 individuals needing health care services for mental health problems. Study participants were randomly selected from an epidemiological survey. Data on help received were collected using the Canadian version of the Perceived Need for Care Questionnaire (PNCQ), and were analyzed using a multinomial logistic regression model. Most help received was in the form of counselling, followed by medication and information. Compared with individuals who received no help, those who reported receiving help for all their needs were more likely to have psychological distress, to be non-verbally aggressive, to consult more healthcare professionals, to be men and to be somewhat older. Compared with individuals who received no help, those who received partial help were more likely to be not addicted to drugs or alcohol, to consult more healthcare professionals, and to be older. Healthcare services should prioritize strategies (e.g., early detection, outreach, public education on mental and addiction disorders) that address barriers to help seeking among youth, as well as individuals addicted to drugs and alcohol or those presenting with aggressive behavior.

## 1. Introduction

A recent systematic review and meta-analysis of 174 surveys carried out between 1980 and 2013 across 63 countries estimated the prevalence of common mental disorders as 17.6% for the general population for a 12-month period, and 29.2% at lifetime.[1] Only a minority of individuals with common mental disorders (between 33% and 46%) seeks help from healthcare professionals [2,3,4,5,6]. According to some epidemiological studies, nearly half of individuals using healthcare services for mental health reasons had not been diagnosed with a mental disorder in the previous year [7,8]. These studies suggest that factors other than the mental disorder diagnosis are at play when individuals seek mental health services [9]. The concept of needing help in the form of mental health treatment is thus not the same as “having” a mental disorder, for which an individual may or may not seek help [10].

In epidemiological studies, help received is not equivalent to healthcare service utilization, which usually refers to professional services (e.g., family physicians, psychiatrists, psychologists, other health professionals, hospitals). Some studies have also included non-professional services, such as self-help groups or personal services (e.g., peer support, spiritual counsellor) [6,11,12]. Help received may take the form of information (about problems, treatments or services), medication, counselling, therapy, guidance regarding personal relationships, and other mental health services (skills training, practical support, referral, *etc.*), regardless of the source of such help [13,14]. In addition, help received does not equate with met needs. It is only when an individual asks for, and receives, the required help that a need is met. A need may be partially met if a person does receive initial help but fails to obtain all or part of any further assistance requested. Finally, a need will obviously not be met if a first request for help goes unheeded [9].

Among previous epidemiological surveys, some have focused on variables related to the perceived needs for mental health care, while others have primarily addressed met and unmet needs [9,10,13,14,15,16,17,18,19,20,21,22,23,24], mental health service utilization [6,11,12,25,26], or barriers to healthcare services [15,26,27,28]. Most studies considered help received as a determinant of unmet needs or patient outcomes, and not as the main variable of interest [13,29]. Help received is an important step in identifying the problem and accessing appropriate services [30]. The one study that has compared unmet, partially and fully met needs [9] overlooks the possible determinants of unmet needs that may be linked to help received. These include variables commonly studied in healthcare service utilization studies, such as beliefs, behavior, perceptions of neighborhood, and professional consultation [31,32,33]. To the best of our knowledge, no previous study has compared help partially received, fully received, or not received at all. Finally, few articles have compared the overall characteristics of treated and non-treated patients. 

Based on the Andersen behavioral model [34], this study conducted with 2332 participants from a Canadian epidemiological catchment area, sought to identify variables associated with help received by 571 individuals who felt they needed help for their mental health, or assistance in dealing with alcohol, drug or emotional problems. Andersen’s model is one of the most widely applied in studies related to health service utilization, unmet needs or patient outcomes, using a broad spectrum of predisposing, need and enabling factors to analyze help received. Predisposing factors include individual characteristics such as age, gender, civil status and self-perceived health. Need factors include health related variables such as number and type of disorders. Finally, enabling factors include variables such as income, social support and neighborhood characteristics. Among the neighborhood characteristics, community participation and collective efficacy are elements that reflect the level of resident collaboration and empowerment in the face of issues such as violence and insecurity. Resident collaboration may in turn have an impact on the appropriateness of help provided to individuals in need. 

## 2. Methods

### 2.1. Study Design and Setting

The research stemmed from the third wave of a longitudinal study in an epidemiological catchment area in Montreal, Canada’s second-largest city. The catchment area had a population of 269,720 within four neighborhoods. This setting included a high ratio of individuals with low incomes (33%**)** and psychological distress. The catchment area included various services, mainly in healthcare (health and social service centers, general practitioners) and mental healthcare (university mental health institute, community-based organizations, private psychologists). 

### 2.2. Selection Criteria and Survey Sample

In order to be included in the study, individuals had to be between 15 and 65 years old and reside in the catchment area. Participants were fully informed about the study and agreed in writing to take part. A university mental health institute ethics board approved the research. 

At baseline (T1: June 2007 to December 2008), 2433 individuals took part in the survey. All were contacted for a second interview (T2) between June 2009 and December 2010. At T2, 1823 responded for a retention rate of 74.9%. At T3 (January 2012 to July 2013), 2334 individuals were interviewed, *i.e.*, 1305 from the T2 cohort and an additional 1029 newly enrolled participants. From the 1823 participants at T2, 518 were lost for the following reasons: 236 had moved away from the study area, 137 could not be reached, 133 refused to participate, 10 had died, and two could not complete the interviews for clinical or psychological reasons. The retention rate at T3 was 72%, which is comparable to that of other epidemiological studies (69% to 76%) after two years or longer [35,36]. The sampling strategy and data collection (T1 and T2) have been described in detail in related publications [33,37]. 

### 2.3. Variables and Measurement Instruments 

The dependent variable, measured at T3, was “help received in the prior 12 months for information, medication, counselling and other perceived needs.” The question read as follows: “Did you receive the help you needed in the past 12 months for information? …for medication? …for counseling or therapy? …for other needs?” Those who reported receiving no help at all were included in the category “help not received”. Those who reported receiving help in all categories of need were included in the category “help fully received”. Those who did not receive help for at least one category of need were included in the category “help partially received”. The dependent variable was then constructed based on the three categories, as follows: “no help received” despite having expressed some needs, “help partially received” (for only some needs such as information, or medication, or counselling, but not for all of them), and “help fully received” (for all needs combined, including information, medication, counselling and other needs). The Canadian version of the Perceived Need for Care Questionnaire (PNCQ; Kappa statistics: .60 for inter-rater reliability) [23,38,39] was used to assess help received by individuals who had perceived needs in terms of their mental health, or in relation to alcohol, drug or emotional problems. This questionnaire identifies the type of help received for expressed needs in the previous 12 months in terms of information, counselling, medication, and other. It also evaluates whether help was adequate (needs totally met) or not (partially met needs, and unmet needs), and identifies the main reasons why help was judged inadequate (e.g., cost, stigma). The sections on adequacy of help and reasons for judging help inadequate was not used in this study.

**Figure 1:**
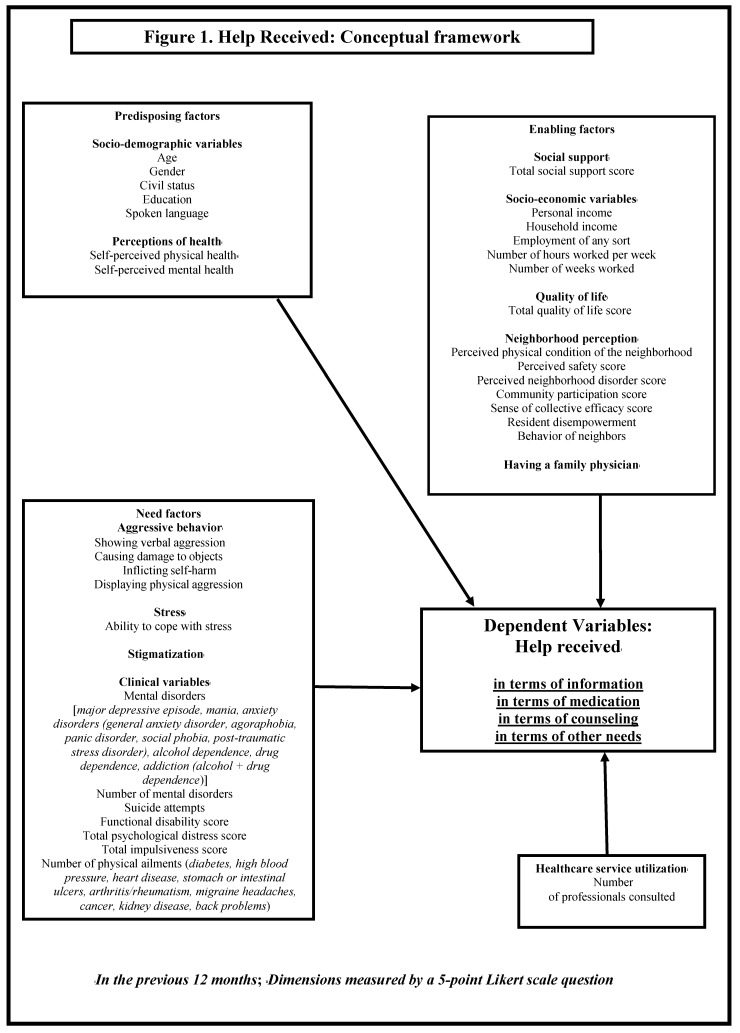
Help received: conceptual framework.

Independent variables measured at T3 were based on the Andersen behavioral model [34]. The independent variables were grouped under predisposing, need and enabling factors, and service utilization variables (Figure 1). The selection of the independent variables was also grounded in the literature review on determinants of mental health service utilization or unmet needs [24,31,33,40]. The concept of service utilization differs significantly from the dependent variable in the present study, “help received when patients reported needs”, and is considered an independent variable in the Andersen model. No collinearity was found between “service utilization” and “help received” in a statistical comparison of the two concepts. Socio-demographic and socio-economic variables (e.g., age, income) were measured using specific questions derived from the CCHS 1.2 questionnaire; [38] and quality of life was assessed using the Satisfaction with Life Domains Scale [41]. In order to assess need factors, the following four instruments were used: the Composite International Diagnostic Interview (CIDI) [42] for mental disorders; the CIDI-SF [42,43] for alcohol and drug dependence; the K-10 Scale [44] for psychological distress; and for impulsivity, the Barratt Impulsivity Scale [45]. 

Stress and stress management strategies were evaluated based on items selected from three scales: the Coping Strategies Indicator (CSI), the Ways of Coping-Revised (WOC-R) tool, and the Critical Survey of Coping Instruments (COPE) [38,46,47]. Aggressive behavior was measured using the Modified Overt Aggression Scale (MOAS) [48]. Enabling factors, included social support, which was evaluated using the Social Provision Scale (SPS) [49], and perceptions of neighborhood, which was measured using six different instruments: the Neighborhood Disorder Scale (NDS), the Neighborhood Physical Condition Scale (NPCS) [50], the Sense of Community Index (SCI) [51], the Community Involvement Scale (CIS) [52], the Sense of Collective Efficacy Scale (SCES) [53], and the Resident Disempowerment Scale (RDS) [50]. Finally, the Mental Health Services Questionnaire (MHSQ) adapted from the CCHS 1.2 questionnaire [38,54], which measures number of hospitalizations and professionals consulted (general practitioners, psychiatrists, psychologists, *etc.*) was used to rate service utilization. 

### 2.4. Analyses

Univariate, bivariate and multivariate analyses were carried out. Univariate analyses consisted of frequency distributions for categorical variables; and mean values, with standard deviations, for continuous variables. A multinomial logistic model was used, as more than two category-dependent variables were assessed at the same time. The multinomial analyses were applied by dividing the sample into three mutually exclusive groups: “no help received” (for any need), “help partially received” (for specific needs only), and “help totally received” (for all needs). Bivariate analyses used binary multinomial logistic regression to assess variables associated with the three-category dependent variables. Variables significantly associated with the dependent variable in the bivariate analyses (chi-square or *t*-test) level (Alpha value set at *p* = 0.10) were used to build the multinomial logistic regression model, based on the backward elimination likelihood ratio technique, with the Alpha value set at *p* = 0.05. Since the variables for building the multinomial model were selected from bivariate analysis without considering the possibility of confounding by other variables, the final model could omit important predictors of perceived need for care that were correlated with other variables. In order to avoid this problem, we added and tested the variables that were non-significant in bivariate analyses (but identified in the literature as important in relation to help received) one by one in the multinomial logistic regression model. The goodness-of-fit was determined using the likelihood ratio and chi-square tests, and the total variance explained was computed using Nagelkerke R^2^. 

## 3. Results 

Of the 2332 participants interviewed at T3, 571 (24%) reported that they had expressed specific needs (addressed or not) and were selected for subsequent study. Among them, 93 (16.3%) stated that they had received help that met all their needs; 374 (65.5%) deemed the help received as partially responding to their needs, while 104 (18.2%) said they had received no help at all.

Table 1 displays characteristics of individuals who received help in the form of information, medication and counselling, in terms of predisposing, enabling and need factors, as well as patterns of health care service utilization. The proportion of participants who stated that they had received counselling help was 49%, whereas 47% were given medication, and 40% obtained information. The mean age was 43 years. Women represented 68% of the sample. Older participants reported having received help mainly for medication, *vs.* help with information and counselling, which were mainly reported by younger participants. The majority of participants were single (66%), with high school education or more (82%). Thirty-nine percent rated their physical health as “fair or poor”, while 42% gave a fair or poor rating for their mental health. Seventy percent of participants had a family doctor. The most prevalent mental health disease among participants was major depression (21%).

Table 2 compares individuals who reported having received full or partial help, with those who reported having received no help at all. The first three columns present frequency distributions, while the two last columns present comparison analyses using a bivariate multinomial analysis. In the bivariate multinomial analysis, each dependent variable was assessed separately for its association with the three-category dependent variable, the reference group being “Help needed but not received”. As compared to the latter group, individuals most likely to report having received partial help were older (predisposing factor: age: 43 *vs.* 39); they had a family doctor (enabling factor: 75% *vs.* 49%), and were less likely to report verbal aggression and addiction to drugs and alcohol (54% *vs.* 65%, 5% *vs.* 13% respectively). They also reported a higher number of physical disorders (need factors: 1.2 *vs.* 0.8), and consulted more professionals (healthcare service utilization: number of professionals consulted: 1.7 *vs.* 1.3). 

Comparing individuals who reported receiving no help with those who felt that the help was fully received, participants in the latter group were more likely to be older (age: 43 *vs.* 39), and less likely to report excellent or very good self-perceived mental health (predisposing factors: 12% *vs.* 14%). Most had a family doctor (enabling factor: 74% *vs.* 49%); and they were less likely to report engaging in verbal aggression or damage to objects (need factors: 50% *vs.* 65%, 23% *vs.* 38% respectively). They were also more likely to have major depressive episodes (38% *vs.* 21%), panic disorder (10% *vs.* 2%), any mental disorder (40% *vs.* 23%), or psychological distress (scores: 15 *vs.* 12%), as well as a higher number of physical and mental disorders (need factors: 1.3 *vs.* 0.8, 0.5 *vs.* 0.3 respectively). Finally, they had consulted a higher number of professionals (healthcare service utilization: 4 *vs.* 1).

A multinomial logistic regression model of variables independently associated with help received in terms of information, medication, counseling and other needs is displayed in Table 3. Compared with individuals who reported having received no help of any kind for their needs, those who received some help were more likely to be older (predisposing factor; Beta: 0.023, *p* = 0.017), with a history of addiction to drug and alcohol (need factor; Beta: 1.344, *p* = 0.004), and to have consulted a greater number of healthcare professionals in the 12 months prior to the study (healthcare service utilization; Beta: 0.803, *p* < 0.001). 

Those who reported receiving help for all their needs compared with participants who had failed to receive any help for their needs, were more likely to be: men (Beta: 0.798, *p* = 0.027), and older (marginal association; predisposing factors, Beta: 0.023, *p* = 0.071); to have experienced higher levels of psychological distress (Beta: 0.048, *p* = 0.047), to have displayed verbal aggression (need factors, Beta: 0.822, *p* = 0.016), and to have met with more healthcare professionals over the previous year (healthcare service utilization, Beta: 1.182, *p* < 0.001). Finally, compared with individuals receiving some help, those receiving help for all their needs were more likely to be men, younger (marginally; predisposing factors); to be affected by psychological distress and to have displayed verbal aggression in the previous year. By contrast, they were less likely to have a history of addiction to drugs and alcohol (need factors). With the exception of scores on the number of healthcare professional consulted in the 12 months prior to the study (healthcare service utilization), which did not differ between the two groups, the significant independent variables were thus in the opposition for individual receiving some help and those receiving help for all their needs. This model explains 32% of the total variance.

**Table 1:** Characteristics of participants who received help in terms of information, medication and counselling (N = 571).

Variables				Total Sample	Information Help	Medication Help	Counselling Help
				(N = 571)	Help Received (N = 229)	Help Received (N = 269)	Help Received (N = 277)
				N (%) Mean (SD)	N (%) Mean (SD)	N (%) Mean (SD)	N (%) Mean (SD)
**Predisposing factors**	Socio-demographic	Age [Mean (SD)]		42.6 (13.4)	41.1 (12.8)	46.0 (12.7)	42.3 (12.7)
		Gender [N (%)]	Women	386 (67.6)	151 (65.9)	180 (66.9)	197 (71.1)
			Men	185 (32.4)	78 (34.1)	89 (33.1)	80 (28.9)
		Civil status [N (%)]	Single	374 (65.5)	149 (65.1)	186 (69.1)	182 (65.7)
			In a conjugal relationship	197 (34.5)	80 (34.9)	83 (30.9)	95 (34.3)
		Education [N (%)]	Less than high school	101 (17.7)	33 (14.4)	55 (20.4)	44 (15.9)
			High school and above	470 (82.3)	196 (85.6)	214 (79.6)	233 (84.1)
	Perceptions of health **^i^**	Self-perceived physical health [N (%)]	Poor or fair	223 (39.1)	90 (39.3)	123 (45.7)	103 (37.2)
			Good	201 (35.2)	79 (34.5)	90 (33.5)	98 (35.4)
			Excellent or very good	147 (25.7)	60 (26.2)	56 (20.8)	76 (27.4)
		Self-perceived mental health [N (%)]	Poor or fair	239 (41.9)	110 (48.0)	134 (49.8)	131 (47.3)
			Good	198 (34.7)	70 (30.6)	84 (31.2)	82 (29.6)
			Excellent or very good	134 (23.5)	49 (21.4)	51 (19.0)	64 (23.1)
**Enabling factors**	Socio-economic [Mean (SD)]	Income	Household income	55,583.3 (45,704.6)	55,702.6 (44,487.7)	49,110.8 (45,112.5)	57,878.6 (47,859.5)
			Personal income	32,413.1 (26,918.4)	34,692.6 (30,336.4)	31,165.0 (28,047.8)	34,939.9 (30,792.6)
		Total social support score		79 (9.9)	79 (9.7)	77.4 (10.4)	78.9 (10.4)
		Total quality of life score		101.6 (15.7)	100.6 (15.3)	99.6 (16.7)	100.9 (16.7)
	Have a family doctor **^i^** [N (%)]			401 (70.2)	172 (75.1)	208 (77.3)	208 (75.1)
	Perception of neighborhood [Mean (SD)]	Perceived physical condition of neighborhood **^a^**		44.6 (11.0)	45.8 (10.1)	44.6 (11.6)	44.0 (11.3)
		Perceived safety **^b^**		3.7 (1.4)	3.6 (1.4)	3.9 (1.4)	3.7 (1.4)
		Perceived disorder in neighborhood **^c^**		42.1 (21.4)	41.4 (20.9)	42.4 (21.7)	44.2 (22.0)
		Community participation score **^d^**		0.9 (1.1)	1.0 (1.1)	0.8 (1.1)	0.9 (1.1)
		Sense of collective efficacy **^e^**		34.4 (6.9)	34.2 (7.1)	34.1 (7.1)	34.1 (7.2)
		Resident disempowerment **^f^**		12.4 (5.9)	12.3 (5.9)	12.9 (6.3)	12.6 (6.3)
		Behavior of neighbors **^g^**		15.2 (9.9)	15.5 (10.2)	15.2 (9.9)	14.9 (9.7)
**Need factors**	Behavior **^i^**	Total impulsiveness score [Mean (SD)] **^h^**		69.3 (5.7)	69.3 (5.3)	69.7 (5.6)	69.2 (5.3)
		Showing verbal aggression [N (%)]		317 (55.5)	127 (55.5)	134 (49.8)	151 (54.5)
		Causing damage to objects [N (%)]		167 (29.2)	72 (31.4)	54 (20.1)	85 (30.7)
		Inflicting self-harm [N (%)]		42 (7.4)	21 (9.2)	19 (7.1)	22 (7.9)
		Displaying physical aggression [N (%)]		52 (9.1)	21 (9.2)	15 (5.6)	31 (11.2)
	Clinical variables **^i^**	Health problems diagnosed [N (%)]	Major depressive episode	117 (20.5)	60 (26.2)	68 (25.3)	68 (24.5)
			Mania	23 (4.0)	10 (4.4)	13 (4.8)	15 (5.4)
			General anxiety disorder	11 (1.9)	6 (2.6)	5 (1.9)	8 (2.9)
			Panic disorder	24 (4.2)	11 (4.8)	17 (6.3)	19 (6.9)
			Social phobia	34 (6.0)	17 (7.4)	17 (6.3)	19 (6.9)
			Agoraphobia	12 (2.1)	7 (3.1)	7 (2.6)	6 (2.2)
			Post-traumatic stress disorder	45 (7.9)	26 (11.4)	32 (11.9)	30 (10.8)
			Alcohol dependence	24 (4.2)	7 (3.1)	6 (2.2)	14 (5.1)
			Drug dependence	25 (4.4)	14 (6.1)	10 (3.7)	15 (5.4)
			Addiction	42 (7.4)	18 (7.9)	15 (5.6)	24 (8.7)
			Any MHD	133 (23.3)	67 (29.3)	74 (27.5)	78 (28.2)
		[Mean (SD)]	Total psychological distress score **^j^**	11.9 (6.9)	12.7 (7.3)	13.5 (7.4)	12.5 (7.2)
			Number of physical illnesses	1.2 (1.4)	1.1 (1.4)	1.5 (1.5)	1.1 (1.4)
			Number of mental disorders	0.3 (0.5)	0.3 (0.5)	0.3 (0.6)	0.3 (0.6)
Healthcare service utilization [Mean (SD)]			Number of professionals consulted	2.6 (1.8)	3.2 (1.8)	3 (1.8)	3.5 (1.8)

Notes: **^i^** In the previous 12 months; **^t^**: ANOVA t test; **^q^**: Chi-square test. Instrument range: **^a^**: 1–110; **^b^**: 2–8; **^c^**: 1–110; **^d^**: 0–1; **^e^**: 1–50; **^f^**: 3–30; **^g^**: 9–45; **^h^**: 30–120; **^j^**: 0–40.

**Table 2:** Characteristics of participants according to level of help received in terms of information, medication and counselling: Bivariate multinomial analyses (N = 571).

Variables				Level of Help (Information, Medication, Counselling and Other)			Bivariate Multinomial Analyses	
				Help Needed But not Received (N = 104)	Help Partially Received (N = 374)	Help Fully Received (N = 93)	Help Partially Received *vs.* no Help Received	Help Fully Received *vs.* no Help Received
				N (%) Mean (SD)	N (%) Mean (SD)	N (% ) Mean (SD)	*p* Value	*p* Value
**Predisposing factors**	Socio-demographic	Age [Mean (SD)]		39.0 (13.5)	43.4 (13.7)	42.9 (11.7)	0.003 **^t^**	0.042 **^t^**
		Gender [N (%)]	Women	70 (67.3)	256 (68.4)	60 (64.5)	0.825 **^q^**	0.680 **^q^**
			Men	34 (32.7)	118 (31.6)	33 (35.5)		
		Civil status [N (%)]	Single	66 (63.5)	245 (65.5)	63 (67.7)	0.699 **^q^**	0.528 **^q^**
			In a conjugal relationship	38 (36.5)	129 (34.5)	30 (32.3)		
		Education [N (%)]	Less than high school	19 (18.3)	68 (18.2)	14 (15.1)	0.984 **^q^**	0.547 **^q^**
			High school and above	85 (81.7)	306 (81.8)	79 (84.9)		
	Perceptions of health **^i^**	Self-perceived physical health [N (%)]	Poor or fair	39 (37.5)	142 (38.0)	42 (45.2)	0.953 **^q^**	0.220 **^q^**
			Good	41 (39.4)	130 (34.8)	30 (32.3)		
			Excellent or very good	24 (23.1)	102 (27.3)	21 (22.6)		
		Self-perceived mental health [N (%)]	Poor or fair	44 (42.3)	133 (35.6)	62 (66.7)	0.058 **^q^**	0.004 **^q^**
			Good	45 (43.3)	133 (35.6)	20 (21.5)		
			Excellent or very good	15 (14.4)	108 (28.9)	11 (11.8)		
**Enabling factors **	Socio-economic [Mean (SD)]	Income	Household income	55108.2	56701.0	51619.8 (51963.8)	0.457 **^t^**	0.420 **^t^**
				(45707.9)	(44091.0)			
			Personal income	29644.7	32557.8	34927.3 (39180.6)		
				(22088.1)	(24294.2)			
		Total social support score		78.6 (9.3)	79.8 (9.9)	76.4 (10.3)	0.270 **^t^**	0.135 **^t^**
		Total quality of life score		100.5 (14.7)	103.1 (15.7)	96.3 (15.7)	0.126 **^t^**	0.071 **^t^**
	Having a family doctor **^i^** [N (%)]			51 (49.0)	281 (75.1)	69 (74.2)	<0.001 **^q^**	<0.001 **^q^**
	Perception of neighborhood [Mean (SD)]	Perceived physical condition of neighborhood **^a^**		44.6 (10,7)	44.5 (10.8)	44.5 (11.8)	0.934 **^t^**	0.948 **^t^**
		Perceived safety **^b^**		3.6 (1.2)	3.7 (1.4)	3.8 (1.5)	0.444 **^t^**	0.418 **^t^**
		Perceived disorder in neighborhood **^c^**		39.9 (22.6)	41.8 (20.7)	45.7 (23)	0.435 **^t^**	0.062 **^t^**
		Community participation score **^d^**		0.8 (11)	0.9 (1)	1 (1.2)	0.773 **^t^**	0.286 **^t^**
		Sense of collective efficacy **^e^**		34.1 (6.6)	34.9 (6.9)	32.9 (7.3)	0.314 **^t^**	0.224 **^t^**
		Resident disempowerment **^f^**		12.2 (5.3)	12.1 (5.9)	13.7 (6.6)	0.965 **^t^**	0.086 **^t^**
		Behavior of neighbors **^g^**		15 (9.9)	15.1 (9.9)	15.5 (9.8)	0.905 **^t^**	0.732 **^t^**
**Need factors **	Behavior **^i^**	Total impulsiveness score [Mean (SD)] **^h^**		69.4 (6.5)	69.1 (5.6)	70.2 (5.0)	0.561 **^t^**	0.334 **^t^**
		Showing verbal aggression [N (%)]		68 (65.4)	203 (54.3)	46 (49.5)	0.044 **^q^**	0.025 **^q^**
		Causing damage to objects [N (%)]		39 (37.5)	107 (28.6)	21 (22.6)	0.083 **^q^**	0.024 **^q^**
		Inflicting self-harm [N (%)]		9 (8.7)	22 (5.9)	11 (11.8)	0.313 **^q^**	0.463 **^q^**
		Displaying physical aggression [N (%)]		12 (11.5)	33 (8.8)	7 (7.5)	0.403 **^q^**	0.344 **^q^**
	Clinical variables **^i^**	Health problems diagnosed [N (%)]	Major depressive episode	22 (21.2)	60 (16.0)	35 (37.6)	0.223 **^q^**	0.012 **^q^**
			Mania	4 (3.8)	13 (3.5)	6 (6.5)	0.857 **^q^**	0.411 **^q^**
			Panic disorder	2 (1.9)	13 (3.5)	9 (9.7)	0.429 **^q^**	0.033 **^q^**
			Social phobia	7 (6.7)	19 (5.1)	8 (8.6)	0.513 **^q^**	0.622 **^q^**
			Addiction	13 (12.5)	17 (4.5)	12 (12.9)	0.004 **^q^**	0.932 **^q^**
			Any mental disorder	24 (23.1)	72 (19.3)	37 (39.8)	0.390 **^q^**	0.012 **^q^**
		[Mean (SD)]	Total psychological distress score **^j^**	12.0 (5.7)	11.1 (6.8)	15.4 (7.3)	0.201 **^t^**	0.001 **^t^**
			Number of physical disorders	0.8 (1.2)	1.2 (1.4)	1.3 (1.4)	0.016 **^t^**	0.019 **^t^**
			Number of mental disorders	0.3 (0.5)	0.2 (0.5)	0.5 (0.6)	0.591 **^t^**	0.015 **^t^**
Healthcare service utilization [Mean (SD)]			Number of professionals consulted	1.3 (1.1)	2.6 (1.7)	3.9 (1.9)	<0.001 **^t^**	<0.001 **^t^**

Notes: **^i^** In the previous 12 months; **^t^**: ANOVA t test; **^q^**: Chi-square test. Instrument range: **^a^**: 1–110; **^b^**: 2–8; **^c^**: 1–110; **^d^**: 0–1; **^e^**: 1–50; **^f^**: 3–30; **^g^**: 9–45; **^h^**: 30-120; **^j^**: 0–40.

**Table 3:** Variables independently associated with level of help received in terms of information, medication, counselling and other needs: Multinomial logistic regression model (Reference category: Help needed but not received; N = 571).

Variable		Level of Help									
		Help Partially Received					Help Fully Received				
		Beta	*p*	OR		95% CI	Beta	*p*	OR		95% CI
				LL	UL				LL	UL	
Predisposing factors	Age	0.023	0.017	1.023	1.004	1.042	0.023	0.071	1.023	0.998	1.049
	Gender (men)	0.425	0.116	1.530	0.900	2.601	0.798	0.027	2.221	1.097	4.499
Need factors	Showing verbal aggression in prior 12 months	0.387	0.132	0.679	0.411	1.123	0.822	0.016	0.439	0.225	0.857
	Psychological distress score	0.027	0.164	0.973	0.937	1.011	0.048	0.047	1.049	1.001	1.101
	Addiction (drug and alcohol) in prior 12 months	1.344	0.004	0.261	0.104	0.651	0.733	0.196	0.481	0.158	1.458
Healthcare service utilization	Number of healthcare professionals consulted in prior 12 months	0.803	<0.001	2.232	1.790	2.783	1.182	<0.001	3.262	2.531	4.204
	Intercept	0.659	0.214				4.231	<0.001			

Notes: Goodness-of-fit: Nagelkerke R^2^: 32.0%; −2 log likelihood: 832.700; Khi-square: 175,578; df: 12; *p* < 0.001; OR: Odds ratio; LL: Lower limit; UL: Upper limit.

## 4. Discussion

With 24% of individuals expressing needs for mental health care, our study is among those reporting the highest rate of perceived needs (between 6% and 32%) [9,19,55,56]. By comparison, 17% of participants in a recent Canadian population study based on the 2012 CCHS-Mental Health stated that they had mental health needs [9]. This difference may be explained by demographic factors that characterize the catchment area. Single individuals were overrepresented in the sample, as were individuals with low income; both are known to be risk factors for psychological distress and mental disorders [57]. The presence of a university mental health institute in the catchment area under study may also explain the greater prevalence of perceived mental health needs, as individuals with mental health disorders tend to live near the services where they receive treatment.

The proportion of individuals who said they had not received any help for their expressed needs (N = 104, 18%) or had received only partial help (N = 374, 65%) confirms that most perceived needs are not fully met, as found previously in most studies related to this subject [10,24,58,59,60,61]. Several reasons might explain this result: individuals may prefer to resolve their own problems or expect that their problems will somehow resolve themselves; individuals may lack access to or be unaware of healthcare services; they may have doubts about the effectiveness of treatment, face financial barriers, or fear being stigmatized, *etc.*[9,19]. 

Consistent with the literature, study participants received more help in the form of counselling, followed by medication and information [13,29,62]. The high ratio of psychologists in the catchment area and in Quebec more generally could explain why a large proportion of individuals in our study received counselling. Psychologists are the second group of professionals most frequently consulted for mental health reasons in Quebec, whereas they rank fourth elsewhere in Canada [63]. The relatively large proportion of participants who received medication could be explained by the fact that medication is free for low income individuals under Quebec’s public drug insurance plan. Information needs are more often self-reported by patients than by their health professionals who tend to deal with information as a secondary issue [64]. Limited consultation time, difficulties experienced by some patients in asking questions, low education, or higher expectations of mental health service availability are other factors that explain insufficient help in the area of mental health [10,65,66]. 

Our model shows that the main difference between those who received no help and those who received full or partial help is explained by need factors (psychological distress, verbal aggression, addiction), predisposing factors (age, gender), and by the number of healthcare professionals consulted. No enabling factor was found to have a significant impact on help received. This finding is supported by the literature, which shows that need factors generally tend to have the strongest influence on individuals in terms of seeking help or using healthcare services. Similarly, in the Grella *et al.* article [56], none of the enabling variables were associated with help seeking. 

Concerning need factors, the association between higher psychological distress and receiving full help is in line with previous studies that have identified strong links between serious psychological distress and variables such as psychiatric treatment [67,68], the number of professionals consulted for mental health reasons [69], chronic physical disorders such as cardiovascular diseases, and early mortality [32,70,71]. By contrast, the Canadian study by Sunderland and Findlay [9] found that psychological distress predicted a greater likelihood that needs would be unmet or only partially met. The link between absence of verbal aggression and full help received seems to imply that professionals may be reluctant to take on patients who display aggressive behavior [72]. Displays of verbal aggression by patients on psychiatric wards are often linked with a history of violence or drug use [73]. Individuals exhibiting aggressive behavior may thus be stigmatized by professionals [74]. Aggressive behavior can also hinder or erode the therapeutic relationship and often contributes to burnout among healthcare providers [75]. Finally, another interesting finding is that individuals with no addiction (drug and alcohol) were more likely to receive partial help, as compared with individuals who received no help. This result is consistent with the research of Sunderland and Findlay [9] on the Canadian population. It confirms that individuals with addiction do not usually seek help for their problems [76] or, alternatively, that they are heavy users of healthcare services without necessarily receiving appropriate care [26,59,77], which in turn depends on the severity of the addiction and whether it is associated with co-occurring mental or physical disorders [78,79,80,81]. Professionals are often reluctant to take on patients with such complex problems [82,83].

With regards to the association between being older and receiving help (predisposing factors), previous studies found that younger individuals were less likely to seek help for mental health reasons [84] and more apt to drop out of treatment once they did [85]. The mean time between the onset of mental disorder and initial treatment is estimated at ten years or more for major depression, anxiety disorders and dependence for the United States, the Netherlands and 15 other countries, according to the World Health Organization (WHO)’s World Mental Health (WMH) surveys [31,86,87,88]. In our study, men were more likely than women to receive all the help they needed. This confirms that help received is not equivalent to healthcare service utilization, as most studies have found that women are more likely to use healthcare services [24,33,89,90,91]. One explanation for this may be that men tend to seek help only after experiencing a sharp deterioration in their mental or physical condition [3,12,92] and then require several types of interventions (medication, counselling, *etc.*). Another possibility is that men tend to use alternative resources (e.g. self-help groups, phone counselling, Internet) which is a fact not usually reported in studies on healthcare service utilization. Meanwhile, women are more likely to seek, and receive help for their mental disorders from general practitioners and from relatives, which could be another reason why they are not getting as much help as men [93]. Furthermore, help received was not associated with civil status (single, married, common-law, *etc.*). However, single individuals are more likely to be affected by a mental health problem compared to individuals living with a spouse or common-law partner. In previous studies on healthcare service utilization, civil status was a controversial variable because it can either be positively or negatively associated with service use [11,94].

Finally, concerning healthcare service utilization, it is logical that help received would increase in relation to the number of professionals consulted. Mental disorders, emotional problems and alcohol and drug problems have multiple causes, requiring various treatments and support from an array of professionals (psychiatrist, psychologist, general practitioner, social worker, *etc.*) [95]. According to the literature, individuals who follow various treatments (e.g., both medication and psychotherapy) with several professionals are more motivated and often have better outcomes [85,96]. 

### Limitations

This study presents some limitations. First, the severity of mental disorders was not considered. Previous studies have reported that the level of disability was a factor in receiving help and in perceiving one’s needs as unmet [18]. Second, this study did not include the entire spectrum of mental disorders, but only affective and anxiety disorders, along with drug and alcohol dependence. Third, due to low numbers of individuals in some subcategories of the sample, some results should be interpreted with caution. Finally, the results reflect population characteristics (e.g. single status, poverty, high prevalence of psychological distress) in the catchment area (Montreal, Canada) and, as such, may not be generalizable to other areas or populations.

## 5. Conclusions 

Many studies have focused on patient needs, mental healthcare service utilization or unmet needs. However, to the best of our knowledge no previous study has attempted to measure help received by individuals who have expressed needs in terms of counselling, information, medication (and other). We believe that the present study uses the most comprehensive set of variables to date, based on the Andersen model, and examines the application of these variables to the help fully received, partially received, or not received in response to needs expressed by individuals. Another strength of this study is that it is grounded in an epidemiological catchment area, and includes key variables not previously considered in related publications using the PNCQ, yet considered important in the healthcare service utilization literature. 

Our findings reveal that younger individuals and those who are addicted or verbally aggressive, are less likely to receive help for their mental or emotional problems or for dependence on drugs or alcohol. Healthcare services should prioritize strategies to eliminate barriers that may prevent such individuals from receiving help. In primary care, early detection of individuals at risk for alcohol or drug dependence may facilitate their access to a healthcare professional before such problems become so acute as to require specialized addiction services. Liaison teams in hospital emergency departments may help identify individuals with addiction, mental disorders or aggressive behaviors, and refer them to appropriate services for their specific needs. Early detection of mental disorders, drug or alcohol addiction, or emotional problems could also be implemented in youth centers or jails. 

Outreach service is another strategy that is particularly effective in spotting youths suffering from mental or addictive disorders and in facilitating their access to healthcare. Public education on substance use and mental disorders, as well as programs especially designed for youth and for individuals with alcohol and drug addiction may reduce this group’s reluctance to seek help from healthcare services. Training on mental health and substance use disorders could provide a basis for dealing more effectively with difficult cases, such as aggressive or addictive behaviors. Finally, equitable access to psychotherapy and counselling may increase the adequacy of help received or services utilization, mainly for single individuals and for those with low income who constitute a major proportion of the population for this catchment area.
